# Brain hypoxia and metabolic crisis are common in patients with acute brain injury despite a normal intracranial pressure

**DOI:** 10.1038/s41598-024-75129-2

**Published:** 2024-10-11

**Authors:** Anton Lund, Anna Forsberg Madsen, Tenna Capion, Helene Ravnholt Jensen, Axel Forsse, John Hauerberg, Sigurður Þor Sigurðsson, Tiit Illimar Mathiesen, Kirsten Møller, Markus Harboe Olsen

**Affiliations:** 1 Copenhagen Neuroanaesthesiology and Neurointensive Care Research Group (CONICA), Department of Neuroanaesthesiology, The Neuroscience Centre, Copenhagen University Hospital – Rigshospitalet, Copenhagen, Denmark; 2 Department of Neurosurgery, The Neuroscience Centre, Copenhagen University Hospital – Rigshospitalet, Copenhagen, Denmark; 3 Department of Clinical Medicine, Faculty of Health and Medical Sciences, University of Copenhagen, Copenhagen, Denmark; 4 Department of Clinical Neuroscience, Karolinska Institutet, Stockholm, Sweden

**Keywords:** Brain hypoxia, Energy Metabolism, Microdialysis, Intracranial pressure, Intracranial hypertension, Oxygen, Subarachnoid hemorrhage, Traumatic brain Injury, Brain injuries, Neurology

## Abstract

Patients with acute brain injury are vulnerable to secondary deterioration, which may go undetected by traditional monitoring. However, multimodal neuromonitoring of brain tissue oxygen tension (PbtO_2_) and energy metabolism may be able to detect such episodes. We report a retrospective, observational study of 94 patients with aneurysmal subarachnoid haemorrhage (SAH) or traumatic brain injury (TBI) who underwent multimodal neuromonitoring during admission. We examined the co-occurrence of pathological neuromonitoring values: elevated intracranial pressure (ICP, > 20 mmHg), inadequate cerebral perfusion pressure (CPP, < 60 mmHg), brain hypoxia (PbtO_2_ < 20 mmHg), and metabolic crisis (lactate/pyruvate ratio > 40 and a glucose level < 0.2 mmol/L in cerebral microdialysate). Mixed effects linear regression demonstrated significant associations between abnormal ICP/CPP, cerebral hypoxia and metabolic crisis. However, brain hypoxia occurred in 40% and 31% of observations in patients with SAH and TBI, respectively, despite normal concurrent values of ICP. Similarly, metabolic crisis was observed in 8% and 16% of measurements for SAH and TBI, respectively, despite a normal ICP. The pattern was identical for CPP. In conclusion, although all neuromonitoring variables are interrelated, brain hypoxia and metabolic crisis are common despite an absence of abnormalities in conventional monitoring. Multimodal neuromonitoring may help identify such episodes and guide individualised treatment.

**Supplementary Information:**

The online version contains supplementary material available at 10.1038/s41598-024-75129-2.

## Introduction

Acute brain injury due to traumatic brain injury (TBI) or aneurysmal subarachnoid haemorrhage (SAH) carries a high morbidity and mortality, and both conditions have a disproportionate societal impact due to the relatively young patient population and large loss of “productive life years”^1,2^. Thus, improving care of these patients is a major global research priority^1^.

The poor prognosis is caused in part by secondary (i.e., delayed) brain injury, which is believed to be at least partly preventable; thus, there is significant emphasis in neurocritical care on protecting the vulnerable brain during the acute phase after injury^3,4^. Neuromonitoring plays an important part in this regard, allowing clinicians to target interventions to prevent secondary insults and maintain brain homeostasis. Accordingly, measurement of intracranial pressure (ICP) and cerebral perfusion pressure (CPP) is widely considered the standard of care in patients with reduced level of consciousness during the initial phase of severe acute brain injury^5,6^. However, so-called multimodal neuromonitoring assessing a combination of different aspects of brain physiology by, e.g., continuous measurement of brain tissue oxygen tension (PbtO_2_) and cerebral microdialysis (CMD) may offer additional information^7^. Accordingly, recent studies have reported that multimodal neuromonitoring may help detect episodes of subclinical cerebral ischaemia which would have gone undetected by traditional ICP/CPP monitoring^8^, and clinical studies of PbtO_2_ monitoring have suggested potential benefits on patient outcome^9,10^. Additionally, some authors have demonstrated that CMD can act as an early warning of delayed cerebral ischaemia (DCI) in patients with SAH, potentially enabling earlier diagnosis and treatment^11,12^.

Discussion remains, however, on how best to combine, interpret and act on the output from multimodal neuromonitoring, and data from large, randomised trials to support its use are still lacking^13,14^. In addition, the techniques are invasive and associated with increased cost, technical challenges both during and after insertion, and potential risks^15^. Thus, there is still a significant gap of knowledge regarding the optimal monitoring strategy in patients with acute brain injury, and the benefits of multimodal neuromonitoring over traditional monitoring are unclear.

The present study examined the relationship between commonly used neuromonitoring modalities (ICP, CPP, PbtO_2_ and cerebral energy metabolism) in a population of neurocritically ill patients, and the influence of these parameters on clinical outcome. We hypothesised that cerebral metabolic crisis and brain hypoxia would be associated with abnormal ICP/CPP, but that they would also commonly occur independently, and that a higher burden of abnormal neuromonitoring values (irrespective of modality) would be associated with unfavourable functional outcome.

## Methods

The study was a retrospective observational study of patients with acute brain injury who underwent multimodal neuromonitoring during admission to the Neurointensive Care Unit (neuro-ICU) at Copenhagen University Hospital – Rigshospitalet, Copenhagen, Denmark between March 2017 and July 2022.

Approval was granted by the Team for Medical Records Research, Centre for Health, The Capital Region of Denmark (ref. R-22020660, 21st of September 2022), and the study was performed in accordance with the 1964 Declaration of Helsinki and its later amendments. Individual patient consent was not required as stated in § 46 (Sects. 2 and 5) of the Danish Health Law (*Sundhedsloven*).

Potentially eligible patients were identified using an internal database of all patients who have undergone multimodal monitoring. Inclusion criteria were as follows:

1. Age ≥ 18 years.
2. Diagnosis of aneurysmal SAH or TBI.
3. Multimodal neuromonitoring during admission (i.e., monitoring of at least two of the following: ICP, PbtO_2_, and/or CMD) and at least 5 available data points for analysis.

### Patient management

Patients were managed according to local protocols based on published recommendations for SAH and TBI^5,6^. For both patient groups this included: (1) intubation and mechanical ventilation in case of a Glasgow Coma Score ≤ 8, or when otherwise clinically indicated; (2) targets for arterial partial pressure of oxygen (PaO_2_) and carbon dioxide (PaCO_2_) of 10–12 kPa and 4.7–5.5 kPa, respectively; (3) maintenance of normothermia; (4) glycaemic control with a blood glucose target of 6–10 mmol/L; (5) low-molecular weight heparin and compression stockings for thromboprophylaxis; (6) maintenance of a CPP > 60 or mean arterial pressure (MAP) > 80 mmHg, and (7) maintenance of an ICP < 20 mmHg through one or more of the following means: 30° head-of bed elevation, increased sedation, hypertonic saline, neuromuscular blockade, and/or surgical intervention such as external ventricular drainage (EVD), haematoma removal, or decompressive craniectomy.

In all patients, computed tomography (CT) scans of the brain were performed upon admission and when otherwise clinically indicated, and an external ventricular drain was inserted in patients with hydrocephalus or when needed to alleviate an elevated ICP.

For SAH, recommendations also included: (1) a systolic blood pressure < 160 mmHg before closure of the aneurysm, and (2) oral administration of nimodipine (60 mg x 6 daily for 21 days). In case of DCI^16^, induced hypertension and/or intraarterial spasmolysis was attempted. The diagnostic workup for patients with SAH also included CT angiography and (if needed) digital subtraction angiography of the brain. These patients underwent endovascular or surgical treatment of the culprit aneurysm within 48 h of admission.

## Multimodal neuromonitoring

ICP was measured using either a Codman^®^ ICP Express^®^ monitor with an intraparenchymal transducer (Integra Lifesciences, NJ, US) inserted either through a triple bolt or directly through a burr hole, or a Spiegelberg^®^ ICP Monitor (Spiegelberg, Hamburg, Germany) with an intraparenchymal transducer integrated into an EVD catheter.

PbtO_2_ was measured using a Licox^®^ PbtO_2_ monitor (Integra Lifesciences, NJ, US) connected to the oxygen probe of the Licox^®^ Brain Tissue Oxygen Monitor Complete Probe Kit (either IM3EU or IM2EU). Insertion of the probe is described below.

Brain metabolism was monitored by CMD using the 70 Microdialysis Bolt Catheter (M Dialysis AB, Stockholm, Sweden) with a 10 × 0.6 mm membrane (length × diameter) and a pore size of 20 kDa. This was perfused with sterile, room-temperature perfusate at a flow rate of 0.3 µL/min. (106 Microdialysis Pump, M Dialysis AB, Stockholm, Sweden). Microdialysate samples were collected every 60 min and analysed for levels of glucose, lactate and pyruvate using a point-of-care analysis system (ISCUS^flex^, M Dialysis AB, Stockholm, Sweden).

Insertion of multimodal neuromonitoring was recommended by local guidelines for all patients with severe SAH or TBI with an initial Glasgow Coma Scale (GCS) score of ≤ 8 and was ultimately done at the discretion of the responsible neurosurgeon. Intracranial monitoring was inserted either in the operating theatre or (less commonly) bedside in the neuro-ICU. In patients with TBI, the monitoring was placed pre-coronally in the least injured hemisphere, whereas in patients with SAH, the monitoring was placed ipsilateral to the aneurysm, or on the left side in the case of midline aneurysms. In all cases, an effort was made to avoid the midline and eloquent areas. The PbtO_2_ probe and CMD catheter were inserted through a double or triple lumen intracranial bolt, with the CMD catheter inserted first due to its fragility.

## Data collection

ICP and PbtO_2_ were continuously monitored using commercially available products as described above, alongside standard physiological parameters. Data were integrated using a patient monitoring system (Intellivue Patient Monitor, Philips, Amsterdam, The Netherlands) and automatically uploaded to the electronic medical record (Sundhedsplatformen, Epic Systems Corporation, WI, US), from which ICP, CPP, PbtO_2_, and MAP were extracted as 15-minute averages. CMD data were extracted directly from the point-of-care analysis system described above as hourly values. These data points were compared to the other variables by averaging the two 15-minute values of the physiological variable on each side of the CMD data point. Prior to analysis, filters were applied to remove obviously erroneous values, i.e.: ICP > 100 or < -5, PbtO2 > 100 or < 2, MAP > 200 or < 1, lactate ≤ 0, pyruvate ≤ 0, and urea < 1. In addition, we removed the first two hours of PbtO_2_-monitoring for each patient to allow for probe equilibration.

Brain tissue oxygenation was categorised as either normal (PbtO_2_ > 20 mmHg), mild hypoxia (PbtO_2_ 15–20 mmHg), or severe hypoxia (PbtO_2_ < 15 mmHg). Metabolic crisis was defined as an LPR > 40 with a brain glucose of < 0.2 mmol/L in cerebral microdialysate^17^. An abnormal ICP was defined as > 20 mmHg, and an abnormal CPP as < 60 mmHg.

Demographic variables and clinical characteristics of the patients were collected from the electronic medical record. Functional outcome was assessed at 6 months after discharge using the modified Rankin Scale (mRS) by review of patient charts. Each score was dichotomised into either a favourable (mRS 0–2) or unfavourable outcome (mRS 3–6).

### Statistical analysis

The nature of our data (pooled, non-independent observations) did not allow for traditional significance testing, and data are therefore mainly presented descriptively. However, to obtain a quantitative estimate of the co-occurrence of neuromonitoring abnormalities and the associated uncertainty, we performed a mixed effects linear regression with neuromonitoring values as fixed effects (dichotomised into normal or abnormal) and patients as random effects to account for within-subject correlation of measurements. Additionally, univariate logistic regressions and the Mann–Whitney U test were used to examine the relationship between neuromonitoring variables (quantified as the percentage of monitoring time with abnormal values) and functional outcome. For exploratory purposes, linear regressions and Pearson’s correlation analyses were also performed between sets of neuromonitoring variables (see Supplementary Fig. S2). Prior to analysis, each neuromonitoring variable was checked for normality and homoscedascity (see Supplementary Fig. S1 for histograms of the raw data). The primary outcome was the incidence of brain hypoxia and metabolic crisis in the presence of elevated ICP and/or reduced CPP. Secondary outcomes were the relationships between each neuromonitoring variable and functional outcome. Data are presented as median (interquartile range) unless otherwise stated, and a P-value < 0.05 was considered statistically significant. All statistical analyses were performed using R (R Core Team, Vienna, Austria).

## Results

Ninety-four patients were included in the study, of which 52 had SAH and 42 had TBI. Patients with SAH underwent a median of 306 (154–392) hours of ICP/CPP-monitoring, 104 (36–258) hours of PbtO_2_-monitoring, and 139 (72–195) hours of valid CMD-monitoring. Patients with TBI underwent a median of 320 (187–417) hours of ICP/CPP-monitoring, 175 (58–261) hours of PbtO_2_-monitoring, and 148 (74–229) hours of valid CMD-monitoring. In general, values were collected continuously with only minor gaps during, e.g., surgery or diagnostic imaging. Demographic and clinical characteristics of the population are described in Table 1.

**Table 1:** Clinical characteristics.

	SAH	TBI	Overall
Number of patients	52	42	94
Male sex, no. (%)	14 (27%)	30 (71%)	44 (47%)
Age, years, median (range)	58 (34–83)	58 (22–86)	58 (22–86)
Initial GCS, median (IQR)	10 (4–14)	6 (3–10)	7 (3–13)
APACHE II score, median (IQR)	20 (18–23)	21 (19–25)	21 (18–23)
WFNS grade, no. (%)			
Grade 1	6 (12%)	–	–
Grade 2	8 (15%)	–	–
Grade 3	2 (4%)	–	–
Grade 4	11 (21%)	–	–
Grade 5	25 (48%)	–	–
Length of stay, days, median (IQR)			
In the ICU	16 (8–28)	22 (11–31.75)	20 (9–31)
In hospital	47 (26–89)	63 (34–91)	57 (28–91)
Six-month outcome, no. (%)			
Favourable (mRS 0–2)	12 (23%)	14 (33%)	26 (28%)
Unfavourable (mRS 3–5)	23 (44%)	19 (45%)	42 (45%)
Dead	16 (31%)	9 (21%)	25 (27%)
Missing data	1 (2%)	0 (0%)	1 (1%)

Demographic and clinical characteristics of the study population as stratified by diagnosis. *APACHE II* Acute Physiology and Chronic Health Evaluation II, *GCS* Glasgow Coma Scale, *ICU* Intensive Care Unit, *IQR* Interquartile range, *mRS* Modified Rankin Scale, *SAH* Subarachnoid haemorrhage, * TBI* Traumatic Brain Injury, *WFNS* World Federation of Neurological Surgeons.

### Interrelationship between neuromonitoring variables

**Fig. 1:**
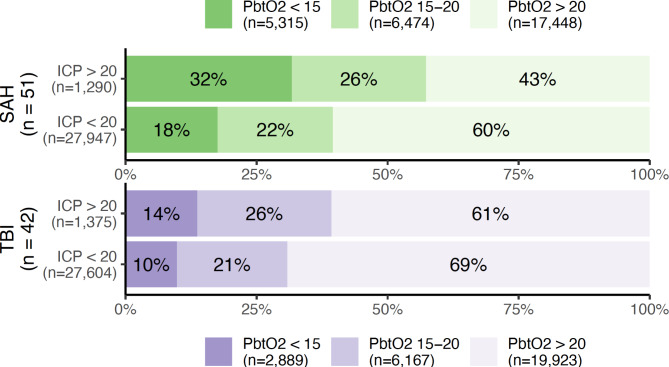
Percentages and absolute numbers of observations with normal (> 20 mmHg), moderately hypoxic (15–20 mmHg), or severely hypoxic (< 15 mmHg) brain tissue oxygen tension (PbtO_2_) as stratified according to the presence or absence of an elevated (> 20 mmHg) intracranial pressure (ICP). Patients with subarachnoid haemorrhage (SAH) and traumatic brain injury (TBI) are illustrated in green and purple, respectively.

The distribution of PbtO_2_ measurements according to the concurrent ICP (dichotomised into normal or abnormal) is shown in Fig. 1. Although both severe and mild brain hypoxia (i.e., a PbtO_2_ < 15 and 15–20 mmHg, respectively) was more common in the presence of intracranial hypertension, severe brain hypoxia was also observed in the presence of a normal concurrent ICP in 18 and 10% of observations in patients with SAH and TBI, respectively, constituting a greater absolute number of observations than those with concurrently abnormal ICP.

**Fig. 2:**
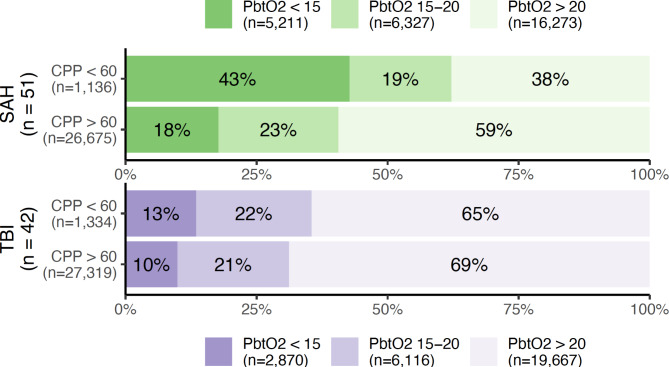
Percentages and absolute numbers of observations with normal (> 20 mmHg), moderately hypoxic (15–20 mmHg), or severely hypoxic (< 15 mmHg) brain tissue oxygen tension (PbtO_2_) as stratified according to the presence or absence of a reduced (< 60 mmHg) cerebral perfusion pressure (CPP). Patients with subarachnoid haemorrhage (SAH) and traumatic brain injury (TBI) are illustrated in green and purple, respectively.

The corresponding relationship between PbtO_2_ and CPP (dichotomised into normal or abnormal) is illustrated in Fig. 2. As above, brain hypoxia was more common in the presence of a reduced CPP, but 41% and 31% of observations with brain hypoxia (for SAH and TBI, respectively) occurred in the presence of a normal CPP.

**Fig. 3:**
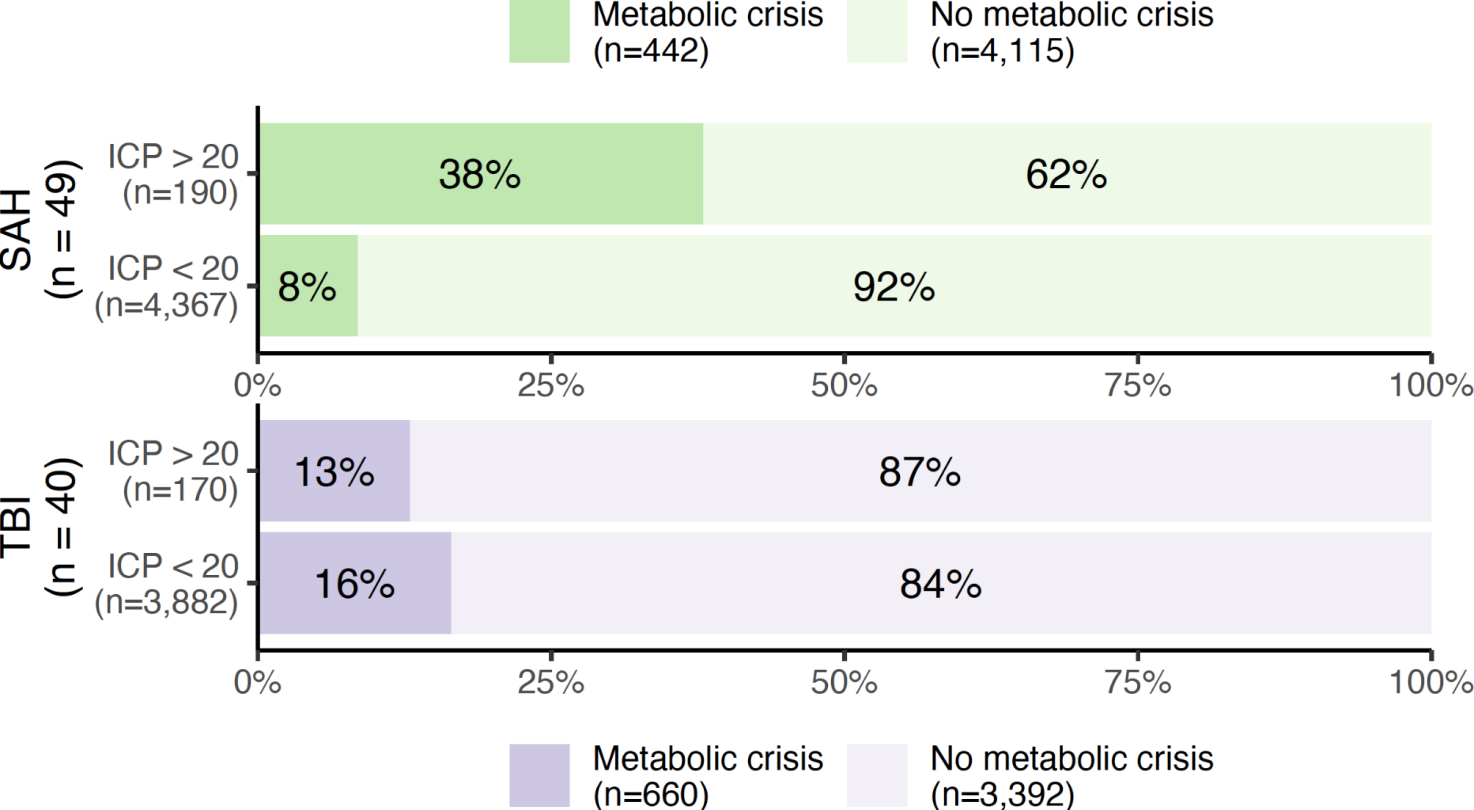
Percentages and absolute numbers of observations with metabolic crisis (a lactate/pyruvate ratio > 40 and glucose < 0.2 mmol/L in cerebral microdialysate) as stratified according to the presence or absence of an elevated (> 20 mmHg) intracranial pressure (ICP). Patients with subarachnoid haemorrhage (SAH) and traumatic brain injury (TBI) are illustrated in green and purple, respectively.

**Fig. 4:**
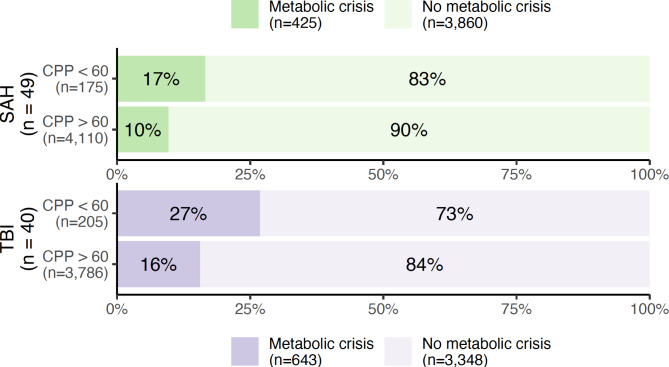
Percentages and absolute numbers of observations with metabolic crisis (a lactate/pyruvate ratio > 40 and glucose < 0.2 mmol/L in cerebral microdialysate) as stratified according to the presence or absence of a reduced (< 60 mmHg) cerebral perfusion pressure (CPP). Patients with subarachnoid haemorrhage (SAH) and traumatic brain injury (TBI) are illustrated in green and purple, respectively.

**Fig. 5:**
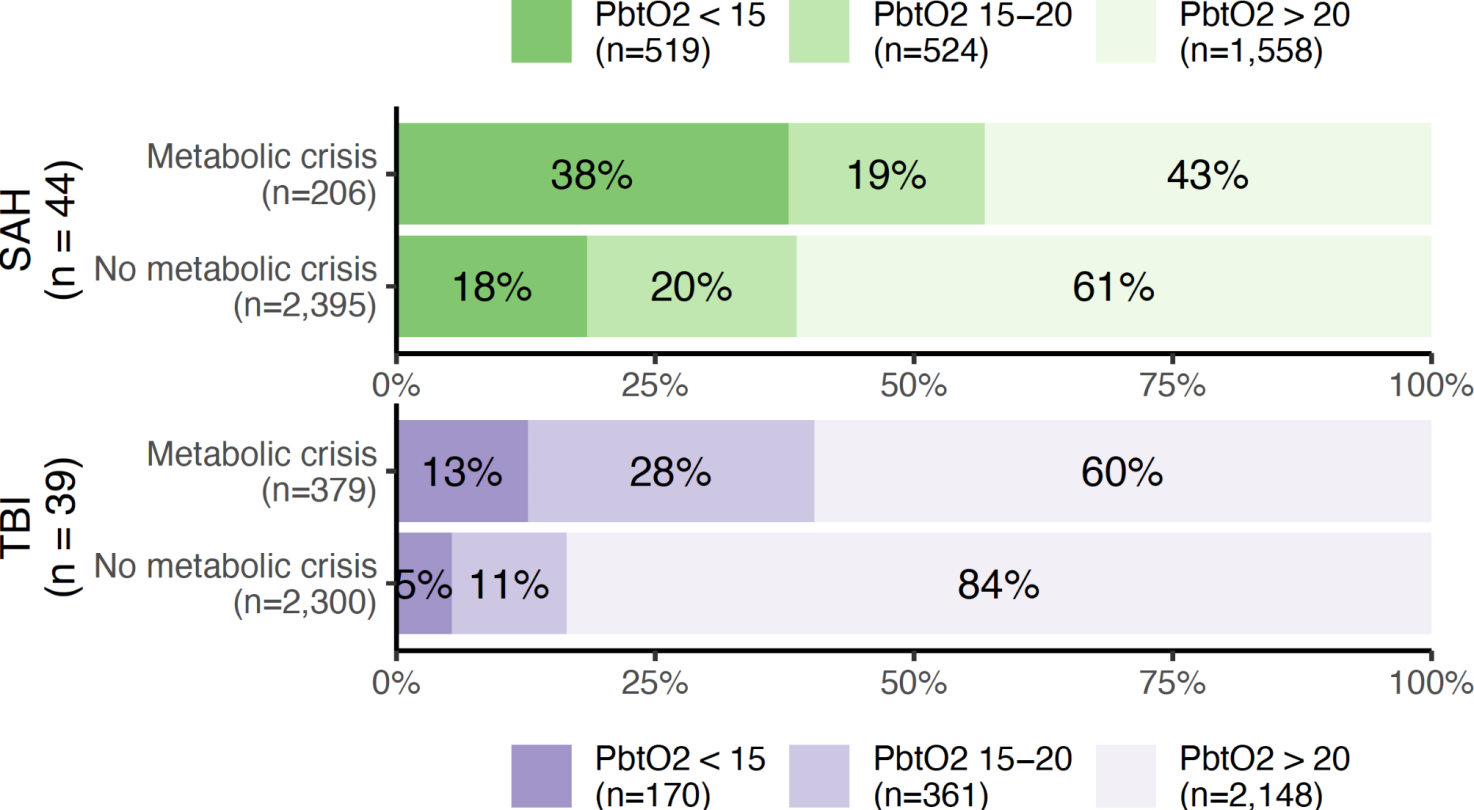
Percentages and absolute numbers of observations with normal (> 20 mmHg), moderately hypoxic (15–20 mmHg), or severely hypoxic (< 15 mmHg) brain tissue oxygen tension (PbtO_2_) as stratified according to the presence or absence of metabolic crisis (a lactate/pyruvate ratio > 40 and glucose < 0.2 mmol/L in cerebral microdialysate) Patients with subarachnoid haemorrhage (SAH) and traumatic brain injury (TBI) are illustrated in green and purple, respectively.

Metabolic crisis debuted a median of 3.8 days (IQR 1.8–7.2) after initiation of monitoring in SAH patients, and after 6.5 (1.15–8.8) days in TBI patients. The relationship between metabolic crisis, ICP and CPP is shown in Figs. 3 and 4, respectively. Metabolic crisis seemed more common in the presence of an abnormal ICP (except in patients with TBI) and CPP, but was also frequently observed in their absence. The relationship between brain oxygenation and metabolic crisis is illustrated in Fig. 5; in both patient groups, metabolic crisis seemed associated with an increased number of observations with brain hypoxia.

The main findings have been summarised in Supplementary Fig. S3.

Results of the mixed effects linear regression between abnormal neuromonitoring variables are shown in Table 2. Abnormalities in the explanatory variable (ICP or CPP) were generally associated with significantly increased odds of brain hypoxia or metabolic crisis. This held true for all combinations of neuromonitoring variables and in both subpopulations (SAH and TBI), except for the analyses concerning the relationship between ICP and metabolic crisis, where an elevated ICP was associated with lower odds of metabolic crisis in patients with TBI, resulting in no significant effect for the total patient population.

**Table 2:** Co-occurrence of abnormal neuromonitoring variables.

Explanatory variable	Outcome variable	SAH		TBI		Overall	
		OR (95% CI)	*P*-value	OR (95% CI)	*P*-value	OR (95% CI)	*P*-value
ICP > 20 mmHg	Brain hypoxia	1.83 (1.60–2.09)	**< 0.001**	1.26 (1.11–1.43)	**< 0.001**	1.49 (1.36–1.63)	**< 0.001**
CPP < 60 mmHg	Brain hypoxia	3.2 (2.75–3.72)	**< 0.001**	1.5 (1.31–1.73)	**< 0.001**	2.13 (1.93–2.36)	**< 0.001**
ICP > 20 mmHg	Metabolic crisis	8.01 (8.00–8.02)	**< 0.001**	0.34 (0.19–0.59)	**< 0.001**	1.39 (0.96–2.01)	0.08
CPP < 60 mmHg	Metabolic crisis	3.36 (1.79–6.31)	**< 0.001**	2.32 (1.44–3.74)	**< 0.001**	2.65 (1.81–3.88)	**< 0.001**

Mixed effects linear regression of the association between abnormal ICP or CPP as the explanatory variable and brain hypoxia (PbtO_2_ < 20 mmHg) or metabolic crisis as the outcome variable. The OR can be interpreted as the increase in the odds of brain hypoxia or metabolic crisis in each observation, when the explanatory variable (ICP or CPP) is abnormal. *CI* Confidence interval, *CPP* Cerebral perfusion pressure, *ICP* Intracranial pressure, *OR* Odds ratio, *PbtO*_*2*_ Brain tissue oxygen tension, *SAH* Subarachnoid haemorrhage, *TBI* Traumatic brain injury.

## Neuromonitoring and functional outcome

**Fig. 6:**
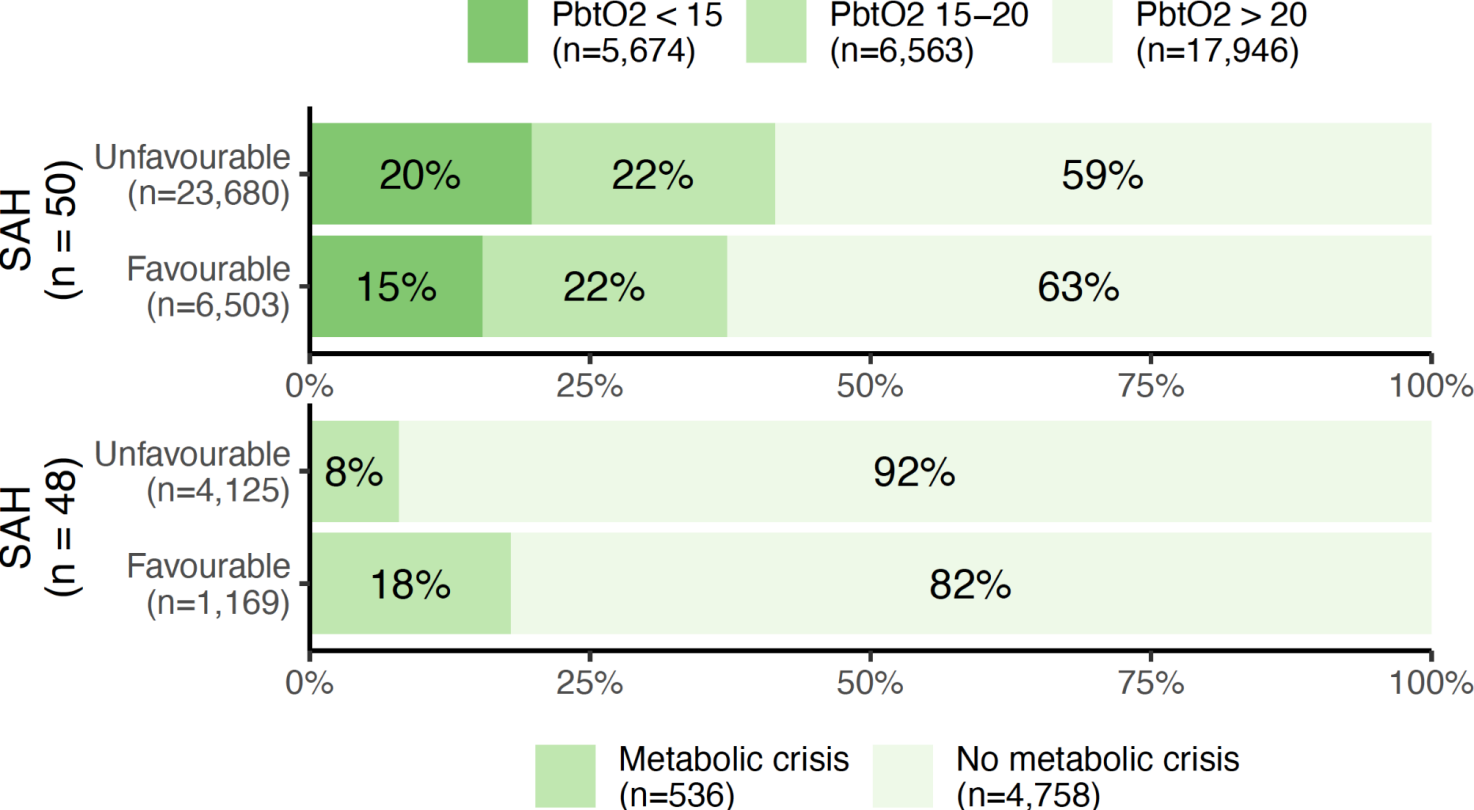
Percentages and absolute numbers of observations with normal (> 20 mmHg), moderately hypoxic (15–20 mmHg), or severely hypoxic (< 15 mmHg) brain tissue oxygen tension (PbtO_2_, upper figure), as well as metabolic crisis (a lactate/pyruvate ratio > 40 and glucose < 0.2 mmol/L in cerebral microdialysate, lower figure) as stratified according to functional outcome in patients with subarachnoid haemorrhage (SAH). A favourable outcome was defined as a modified Rankin Scale of 0–2 at 6 months after ictus, whereas an unfavourable outcome was defined as a score of 3–6.

**Fig. 7:**
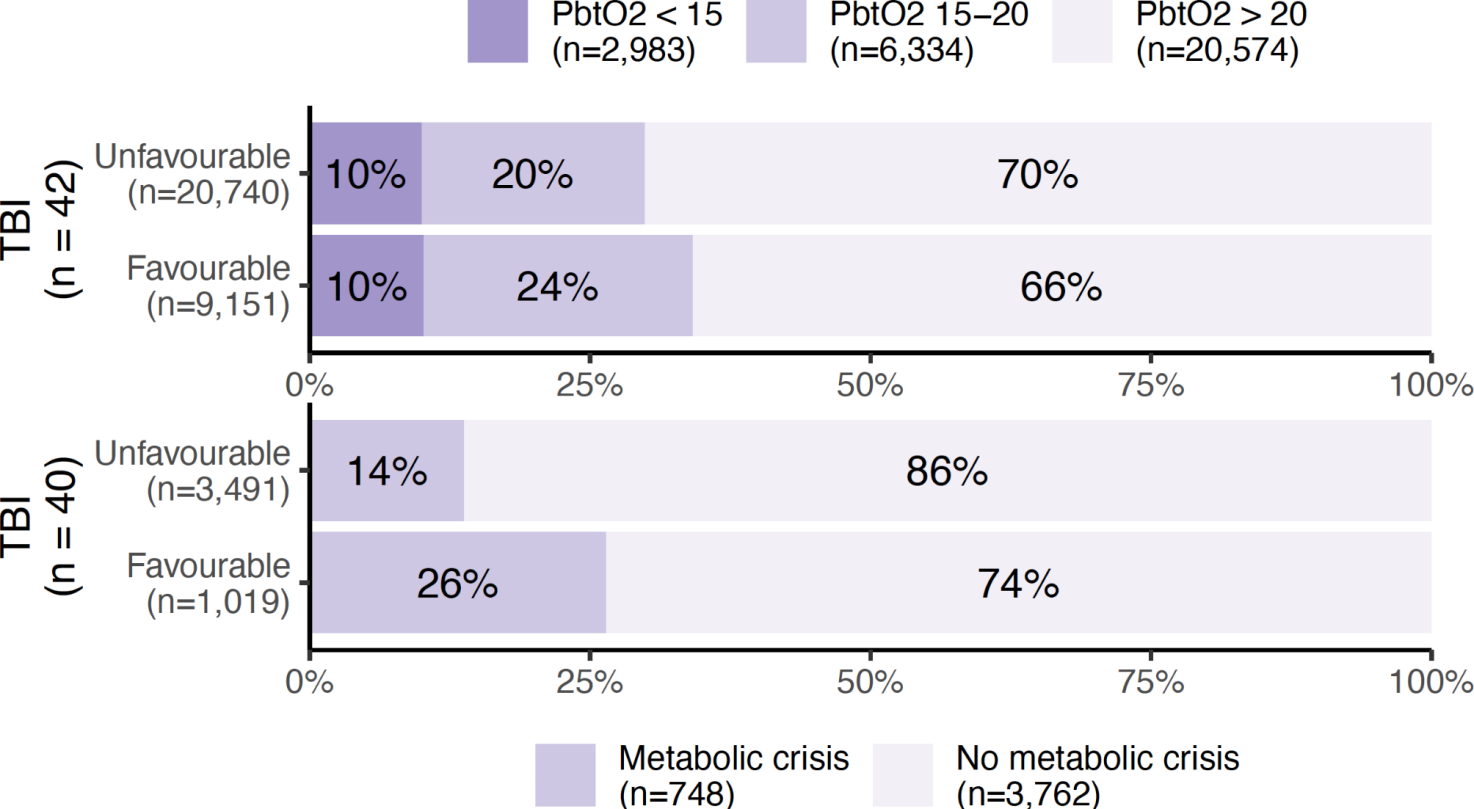
Percentages and absolute numbers of observations with normal (> 20 mmHg), moderately hypoxic (15–20 mmHg), or severely hypoxic (< 15 mmHg) brain tissue oxygen tension (PbtO_2_, upper figure), as well as metabolic crisis (a lactate/pyruvate ratio > 40 and glucose < 0.2 mmol/L in cerebral microdialysate, lower figure) as stratified according to functional outcome in patients with traumatic brain injury (TBI). A favourable outcome was defined as a modified Rankin Scale of 0–2 at 6 months after ictus, whereas an unfavourable outcome was defined as a score of 3–6.

The relationship between functional outcome and the proportion of observations with brain hypoxia and metabolic crisis are illustrated in Fig. 6 (for SAH) and Fig. 7 (for TBI). Interestingly, a favourable outcome seems associated with an increased number of observations with metabolic crisis in both patient groups.

To examine the relationship between the “burden of pathological neuromonitoring observations” and outcome, the percentage of time spent with abnormal values was calculated for each variable in each individual patient. The relationship between this parameter and functional outcome was examined using the Mann–Whitney U test (Table 3), as well as a univariate logistic regression (Table 4). The only variable significantly associated with outcome was PbtO_2_: in the logistic regression analysis, a 1% increase in the time spent with a PbtO_2_ < 20 mmHg increased the odds of an unfavourable outcome by 2% (95% CI 0–4%, *p* = 0.041) in the total patient population. However, this result could not be replicated in each subpopulation (SAH and TBI).

**Table 3:** Abnormal neuromonitoring and outcome.

Neuromonitoring abnormality (as % of monitoring time)	SAH			TBI		
	Favourable outcome (median, IQR)	Unfavourable outcome (median, IQR)	*P*-value	Favourable outcome (median, IQR)	Unfavourable outcome (median, IQR)	*P*-value
ICP > 20 mmHg	1.7 (0.7–3.1) %	1.9 (1.2–5.8) %	0.28	2.6 (1.5–4.6) %	1.6 (1.1–3.4) %	0.32
CPP < 60 mmHg	1.6 (1.4–1.9) %	4.0 (1.3–6.2) %	0.11	6.3 (3.3–9.5) %	4.5 (1.9–5.3) %	0.052
PbtO_2_ < 20 mmHg	26 (1.4–48) %	37 (24–63) %	0.07	16% (8.7–32)	20 (10–43) %	0.65
Metabolic crisis	9.4 (1.7–25) %	3.0 (0.35–25) %	0.67	14 (1.9–23) %	0.91 (0.0–8.4) %	0.12

The percentage of total monitoring time spent with abnormal neuromonitoring values stratified by outcome in each patient group. P-values are for the comparison between outcome strata for each variable (Mann–Whitney U-test). *CPP* Cerebral perfusion pressure, *ICP* Intracranial pressure, *PbtO*_*2*_ Brain tissue oxygen tension, *SAH* Subarachnoid haemorrhage, *TBI* Traumatic brain injury.

**Table 4:** Univariate logistic regressions.

Explanatory variable	SAH		TBI		Overall	
	OR (95% CI)	*P*-value	OR (95% CI)	*P*-value	OR (95% CI)	*P*-value
ICP > 20 mmHg (%)	1.10 (1.00–1.39)	0.202	0.90 (0.71–1.12)	0.337	1.06 (0.99–1.19)	0.221
CPP < 60 mmHg (%)	1.11 (0.98–1.38)	0.248	0.90 (0.75–1.02)	0.134	1.00 (0.94–1.08)	0.955
PbtO_2_ < 20 mmHg (%)	1.03 (1.00–1.06)	0.054	1.01 (0.98–1.04)	0.499	1.02 (1.00–1.04)	**0.041**
Metabolic crisis (%)	1.00 (0.97–1.02)	0.781	0.99 (0.97–1.02)	0.527	0.99 (0.98–1.01)	0.520

Univariate logistic regressions of the relationship between functional outcome and each individual neuromonitoring variable (summarised as the percentage of total monitoring time spent with abnormal values). The OR can be interpreted as an increase in the odds of an unfavourable outcome (i.e., a modified Rankin Scale of 3–6) with each percent increase in the time spent with an abnormal value of the explanatory variable. *CI* Confidence interval, *CPP* Cerebral perfusion pressure, *ICP* Intracranial pressure, *OR* Odds ratio, *PbtO*_*2*_ Brain tissue oxygen tension, *SAH* Subarachnoid haemorrhage, *TBI* Traumatic brain injury.

## Discussion

The present study examined the interrelationship between various neuromonitoring variables in a cohort of patients with acute brain injury (SAH and TBI), as well as the influence on clinical outcome. The principal finding is that abnormalities in ICP and CPP increase the risk of brain hypoxia and metabolic crisis as hypothesised, but these abnormalities also frequently occur independently of one another. However, no clear relationship between the individual burden of pathological neuromonitoring and functional outcome could be established in this population.

Previous studies have found that conventional monitoring of ICP and CPP may be insufficient to detect all episodes of cerebral dyshomeostasis and, conversely, that increased ICP and reduced CPP can also occur when brain oxygenation and metabolism are unaffected^8,18^. Related studies in children with TBI have similarly found that the relationship between ICP and PbtO_2_ is minor^19^, and that brain hypoxia is common even when acceptable treatment targets for ICP, CPP, and systemic oxygenation are achieved^20^. The present study aligns well with the prior literature and suggests that episodes of local ischaemia, or metabolic compromise for other reasons, are frequent even in the absence of pressure abnormalities which could adversely affect perfusion. This is an important finding, as ICP and CPP are treatment targets in most neuro-ICUs, and patients spend extended periods of their admission with these parameters within the normal range; thus, multimodal neuromonitoring could be valuable for detecting latent abnormalities and guiding interventions in patients that would otherwise be considered stable. Whether such interventions also improve patient outcomes is outside the scope of the present work. While the currently available (primarily observational) evidence suggests benefits of PbtO_2_-guided therapy^10,21^, the recently published OXY-TC phase III randomised controlled trial in patients with TBI did not demonstrate any benefits on neurological outcome at 6 months^22^. Other ongoing randomised controlled trials will hopefully clarify the issue further^10,14^.

Interestingly, in our population, patients with SAH appeared to have an overall higher prevalence of abnormal multimodal neuromonitoring values. Potential causes include the placement of the probe ipsilaterally to the aneurysm in patients with SAH and contralaterally to the most severe injuries in patients with TBI. Thus, the probes may have sampled more abnormal tissue in patients with SAH; indeed, prior studies have demonstrated that CMD probe location (i.e., the proximity to focal brain lesions) can greatly affect metabolite measurements^23^. Another potential cause is selection bias; although the clinical recommendation in our centre is for all patients with TBI or SAH and an initial GCS < 9 to undergo multimodal neuromonitoring, in reality, many patients did not undergo this intervention for reasons ranging from coagulopathy to the personal preferences of the neurosurgeon in charge. This may have adversely affected the generalisability of this sample.

In a univariate logistic regression analysis of the total patient population, an association was found between cerebral hypoxia and functional outcome, in agreement with prior studies^24^. This could, however, not be reproduced in the individual subpopulations (TBI and SAH), likely due to a lack of statistical power. Identical analyses for the remaining neuromonitoring parameters did not demonstrate any relationship between abnormal values and outcome. Surprisingly, the illustrations of outcome vs. the prevalence of metabolic crisis (Figs. 6 and 7) give the impression that a higher proportion of measurements with metabolic crisis was associated with a favourable outcome, which conflicts with prior literature on the relationship between CMD and outcome^25^. Similar counterintuitive results were observed in the mixed effects model, where the odds of metabolic crisis were lower when ICP was elevated in the subgroup of patients with TBI (Table 2). There are several potential explanations: Firstly, there could be inherent problems with the definition of “metabolic crisis”, which is an aggregate measure. There is a significant loss of information when 3 continuous variables are simplified into one binary parameter; the established thresholds could be incorrect, or the individual components of the parameter (glucose, lactate, pyruvate, and/or the LPR) could be better predictors by themselves. Secondly, previous studies have found that an elevated lactate and pyruvate simultaneously with a normal PbtO_2_ (i.e., hyperglycolysis) is associated with a better functional outcome in patients with SAH, possibly in part because lactate acts as a substrate for the injured brain^26,27^. Furthermore, metabolic crisis (defined as an LPR > 40) on a non-ischaemic basis is common in patients with TBI and is likely a more benign phenomenon^28^. The present study does not examine lactate, pyruvate, and LPR individually, but this could be an explanation for the conflicting associations between metabolic crisis and outcome observed here.

To summarise, there is no clear connection between abnormal neuromonitoring and patient outcome in this study. This may be due to the sample size, patient selection (as described above), or the way abnormal neuromonitoring is quantified (“percentage of time with abnormal values”)—other approaches (i.e., area under the curve) or alternative threshold values (i.e., ICP > 25 mmHg or PbtO_2_ < 10 mmHg) could have yielded other results. Additionally, in the present work each observation was quantified as a 15-minute average; more sustained abnormalities (e.g., ≥ 1 h) could potentially have a stronger relation to clinical outcome.

### Strengths and limitations

The study has several strengths. First, the study examines a significantly larger population than previous studies of multimodal neuromonitoring examining similar hypotheses^8^. Second, data were collected at a higher temporal resolution using electronic data capture, which eliminates bias associated with traditional manual data collection. Conversely, this may also entail a greater risk of artifacts, which are not automatically identified and removed; the use of data based on 15-minute averages should, however, greatly reduce the influence of individual artefacts, and filters were applied prior to analysis to remove obviously erroneous values. Third, this study examined (and compared) both patients with TBI and SAH, in contrast to prior studies, which have focused more narrowly on single patient groups^8,18^.

However, the study also has its limitations. First, the data were obtained and analysed in a retrospective manner. Second, the study was conducted at a single centre, which may limit the external validity. Third, as discussed above, the population may not have been representative for all patients with severe TBI or SAH admitted to our neuro-ICU. Fourth, the neuromonitoring modalities sample data from a limited volume of brain tissue, which may not reflect the global status of the brain; however, this is a fundamental condition in all similar studies. Furthermore, as mentioned above, the placement of the monitors was different in the two patient groups, as they were placed in the “least injured” side of the brain in patients with TBI and ipsilateral to the source of haemorrhage in patients with SAH. Fifth, patients received targeted interventions for abnormal ICP, CPP, PbtO_2_ and CMD-values as part of their care, which may have confounded the influence of each parameter or diluted their effect on outcome. Sixth, we did not account for any time relation between the variables (e.g., cases where elevated ICP or low CPP preceded brain hypoxia or metabolic crisis); rather, we looked at the simultaneous occurrence of abnormalities. This of course limits our ability to establish causality between the events, although this was not the primary aim of the study. Seventh, the assessment of microdialysis abnormalities in our study was limited to energy metabolites, and the aggregate measure “metabolic crisis” was used, which may have overlooked isolated abnormalities in glucose, pyruvate, or lactate levels. However, a comprehensive examination of the cerebral metabolic status of patients with acute brain injury is outside of the scope of the present work. Eighth, results were largely dichotomised in the present study (i.e., into “good” or “bad” neuromonitoring values or outcome), which unavoidably entails a loss of information. However, the cut-offs used were based on broadly accepted normal ranges, and we believe that this approach facilitates clinical applicability of the results and helps mitigate any skewness in the underlying data. Finally, in a material of this size, the characteristics of individual patients (with, e.g., a high burden of abnormal neuromonitoring) could skew the overall associations; however, there is no clear-cut method of delineating these patients and accounting for them.

## Conclusions

In the present study, conventional ICP and CPP monitoring was unable to detect all episodes of cerebral distress in a population of patients with SAH and TBI, thus supporting a broader approach to monitoring in neurocritical care. However, no clear relationship between the individual burden of abnormal multimodal neuromonitoring values and outcome could be established. Compared to isolated monitoring of ICP and CPP, the combination of several monitoring modalities provides the opportunity to tailor treatment options such as sedation level, mechanical ventilation, targeted temperature management, and osmotherapy to individual patient needs. However, the evidence base for multimodal neuromonitoring in patients with ABI is still limited, and further studies are needed to clarify optimal monitoring combinations and treatment algorithms. Further research should also focus on integrating data from different monitors to identify ideal pathological thresholds, different patterns of cerebral dysfunction, and their clinical and prognostic implications.

## Electronic supplementary material

Below is the link to the electronic supplementary material.

Supplementary Material 1

## Data Availability

The data collected and analysed in the current study are not publicly available due to patient confidentiality considerations but are available from the corresponding author on reasonable request and after relevant regulatory approvals for data transfer are obtained.

## References

[1] Maas, A. I. R. et al. Traumatic brain injury: progress and challenges in prevention, clinical care, and research. *Lancet Neurol. ***21**, 1004–1060 (2022). 36183712 10.1016/S1474-4422(22)00309-X PMC10427240

[2] Rinkel, G. J. & Algra, A. Long-term outcomes of patients with aneurysmal subarachnoid haemorrhage. *Lancet Neurol. ***10**, 349–356 (2011). 21435599 10.1016/S1474-4422(11)70017-5

[3] Stocchetti, N. et al. Neuroprotection in acute brain injury: An up-to-date review. *Crit. Care*. **19**, 186. 10.1186/s13054-015-0887-8 (2015). 10.1186/s13054-015-0887-8 PMC4404577 25896893

[4] Werner, C. & Engelhard, K. Pathophysiology of traumatic brain injury. *Br. J. Anaesth.*. **99**, 4–9. 10.1093/bja/aem131 (2007). 10.1093/bja/aem131 17573392

[5] Carney, N. et al. Guidelines for the management of severe traumatic brain Injury, Fourth Edition. *Neurosurgery*. **80**, 6–15 (2017). 27654000 10.1227/NEU.0000000000001432

[6] Connolly, E. S. et al. Guidelines for the management of aneurysmal subarachnoid hemorrhage: a guideline for healthcare professionals from the American Heart Association/American Stroke Association. *Stroke*. **43**, 1711–1737 (2012). 22556195 10.1161/STR.0b013e3182587839

[7] Rajagopalan, S. & Sarwal, A. Neuromonitoring in critically ill patients. *Crit. Care Med. ***51**, 525–542 (2023). 36794946 10.1097/CCM.0000000000005809

[8] Chen, H. I. et al. Detection of cerebral compromise with multimodality monitoring in patients with subarachnoid hemorrhage. *Neurosurgery*. **69**, 53–63 (2011). 21796073 10.1227/NEU.0b013e3182191451

[9] Okonkwo, D. O. et al. Brain oxygen optimization in severe traumatic brain Injury Phase-II: a phase II randomized trial. *Crit. Care Med. ***45**, 1907–1914 (2017). 29028696 10.1097/CCM.0000000000002619 PMC5679063

[10] Gouvêa Bogossian, E. et al. The impact of invasive brain oxygen pressure guided therapy on the outcome of patients with traumatic Brain Injury: a systematic review and Meta-analysis. *Neurocrit. Care*. **37**, 779–789 (2022). 36180764 10.1007/s12028-022-01613-0

[11] Skjøth-Rasmussen, J., Schulz, M., Kristensen, S. R. & Bjerre, P. Delayed neurological deficits detected by an ischemic pattern in the extracellular cerebral metabolites in patients with aneurysmal subarachnoid hemorrhage. *J. Neurosurg. ***100**, 8–15 (2004). 14743906 10.3171/jns.2004.100.1.0008

[12] Rostami, E. et al. Early low cerebral blood flow and high cerebral lactate: prediction of delayed cerebral ischemia in subarachnoid hemorrhage. *J. Neurosurg. ***128**, 1762–1770 (2018). 28574309 10.3171/2016.11.JNS161140

[13] Le Roux, P. et al. Consensus summary statement of the International Multidisciplinary Consensus Conference on Multimodality Monitoring in Neurocritical Care: a statement for healthcare professionals from the Neurocritical Care Society and the European Society of Intensive Care Medicine. *Intensive Care Med. ***40**, 1189–1209 (2014). 25138226 10.1007/s00134-014-3369-6

[14] Leach, M. R. & Shutter, L. A. How much oxygen for the injured brain – can invasive parenchymal catheters help? *Curr. Opin. Crit. Care*. **27**, 95–102 (2021). 33560016 10.1097/MCC.0000000000000810 PMC7987136

[15] Pease, M., Nwachuku, E., Goldschmidt, E., Elmer, J. & Okonkwo, D. O. Complications from multimodal monitoring do not affect long-term outcomes in severe traumatic brain Injury. *World Neurosurg. ***161**, e109–e117 (2022). 35077890 10.1016/j.wneu.2022.01.059 PMC9081234

[16] Vergouwen, M. D. I. et al. Definition of delayed cerebral ischemia after aneurysmal subarachnoid hemorrhage as an outcome event in clinical trials and observational studies: proposal of a multidisciplinary research group. *Stroke*. **41**, 2391–2395 (2010). 20798370 10.1161/STROKEAHA.110.589275

[17] Hutchinson, P. J. et al. Consensus statement from the 2014 International Microdialysis Forum. *Intensive Care Med. ***41**, 1517–1528 (2015). 26194024 10.1007/s00134-015-3930-y PMC4550654

[18] Oddo, M. et al. Brain hypoxia is associated with short-term outcome after severe traumatic brain injury independently of intracranial hypertension and low cerebral perfusion pressure. *Neurosurgery*. **69**, 1037–1045 (2011). 21673608 10.1227/NEU.0b013e3182287ca7

[19] Rohlwink, U. K. et al. The relationship between intracranial pressure and brain oxygenation in children with severe traumatic brain injury. *Neurosurgery*. **70**, 1220–1230 (2012). 22134142 10.1227/NEU.0b013e318243fc59

[20] Figaji, A. A., Fieggen, A. G., Argent, A. C., Leroux, P. D. & Peter, J. C. Does adherence to treatment targets in children with severe traumatic brain injury avoid brain hypoxia? A brain tissue oxygenation study. *Neurosurgery*. **63**, 83–91 (2008). 18728572 10.1227/01.NEU.0000335074.39728.00

[21] Hays, L. M. C. et al. Effects of brain tissue oxygen (PbtO2) guided management on patient outcomes following severe traumatic brain injury: a systematic review and meta-analysis. *J. Clin. Neurosci. ***99**, 349–358 (2022). 35364437 10.1016/j.jocn.2022.03.017

[22] Payen, J. F. et al. Intracranial pressure monitoring with and without brain tissue oxygen pressure monitoring for severe traumatic brain injury in France (OXY-TC): an open-label, randomised controlled superiority trial. *Lancet Neurol. ***22**, 1005–1014 (2023). 37863590 10.1016/S1474-4422(23)00290-9

[23] Kofler, M. et al. The importance of probe location for the Interpretation of Cerebral Microdialysis Data in Subarachnoid Hemorrhage patients. *Neurocrit. Care*. **32**, 135–144 (2020). 31037640 10.1007/s12028-019-00713-8 PMC7012974

[24] Maloney-Wilensky, E. et al. Brain tissue oxygen and outcome after severe traumatic brain injury: a systematic review. *Crit. Care Med. ***37**, 2057–2063 (2009). 19384213 10.1097/CCM.0b013e3181a009f8

[25] Zeiler, F. A. et al. A systematic review of cerebral microdialysis and outcomes in TBI: relationships to patient functional outcome, neurophysiologic measures, and tissue outcome. *Acta Neurochir. (Wien)*. **159**, 2245–2273 (2017). 28988334 10.1007/s00701-017-3338-2 PMC5686263

[26] Oddo, M. et al. Brain lactate metabolism in humans with subarachnoid hemorrhage. *Stroke*. **43**, 1418–1421 (2012). 22343642 10.1161/STROKEAHA.111.648568

[27] Nordström, C. H. et al. Bedside interpretation of cerebral energy metabolism utilizing microdialysis in neurosurgical and general intensive care. *Front. Neurol. ***13**, 968288 (2022). 36034291 10.3389/fneur.2022.968288 PMC9399721

[28] Vespa, P. et al. Metabolic crisis without brain ischemia is common after traumatic brain injury: a combined microdialysis and positron emission tomography study. *J. Cereb. Blood Flow. Metab*. **25**, 763–774 (2005). 15716852 10.1038/sj.jcbfm.9600073 PMC4347944

