# Supplementary material for: Brain hypoxia and metabolic crisis are common in patients with acute brain injury despite a normal intracranial pressure
Source: Sci Rep. 2024 Oct 11;14:23828. doi: 10.1038/s41598-024-75129-2 (PMC11470048; doi:10.1038/s41598-024-75129-2)
Supplement: Supplementary file 1 — Supplementary Material 1 [file 41598_2024_75129_MOESM1_ESM.pdf]

Brain hypoxia and metabolic crisis are common in patients with acute brain injury despite a normal intracranial pressure:
supplemental material

Anton Lund[1]*, MD; Anna Forsberg Madsen[1]*, MD; Tenna Capion[2], MD; Helene Ravnholt Jensen[1], MSN; Axel Forsse[2], MD, PhD; John Hauerberg[2], MD; Sigurður Þor Sigurðsson[1], MD, PhD; Tiit I. Mathiesen[2,3,4], MD, DMSc; Kirsten Møller[1,3], MD, PhD, DMSc; & Markus Harboe Olsen[1], MD, PhD.

*Co-primary authorship

Affiliations:

[1]Copenhagen Neuroanaesthesiology and Neurointensive Care Research Group (CONICA), Department of Neuroanaesthesiology, The Neuroscience Centre, Copenhagen University Hospital – Rigshospitalet, Copenhagen, Denmark

[2]Department of Neurosurgery, The Neuroscience Centre, Copenhagen University Hospital – Rigshospitalet, Copenhagen, Denmark

[3]Department of Clinical Medicine, Faculty of Health and Medical Sciences, University of Copenhagen, Copenhagen, Denmark

[4]Department of Clinical Neuroscience, Karolinska Institutet, Stockholm, Sweden

Correspondence:

Anton Lund, MD

Department of Neuroanaesthesiology, The Neuroscience Centre

Copenhagen University Hospital – Rigshospitalet

Blegdamsvej 9, DK-2100 Copenhagen, Denmark

Telephone (work): +45 35451616

E-mail (work): anton.lund@regionh.dk

SUPPLEMENTARY FIGURE S1:

HISTOGRAMS OF NEUROMONITORING PARAMETERS

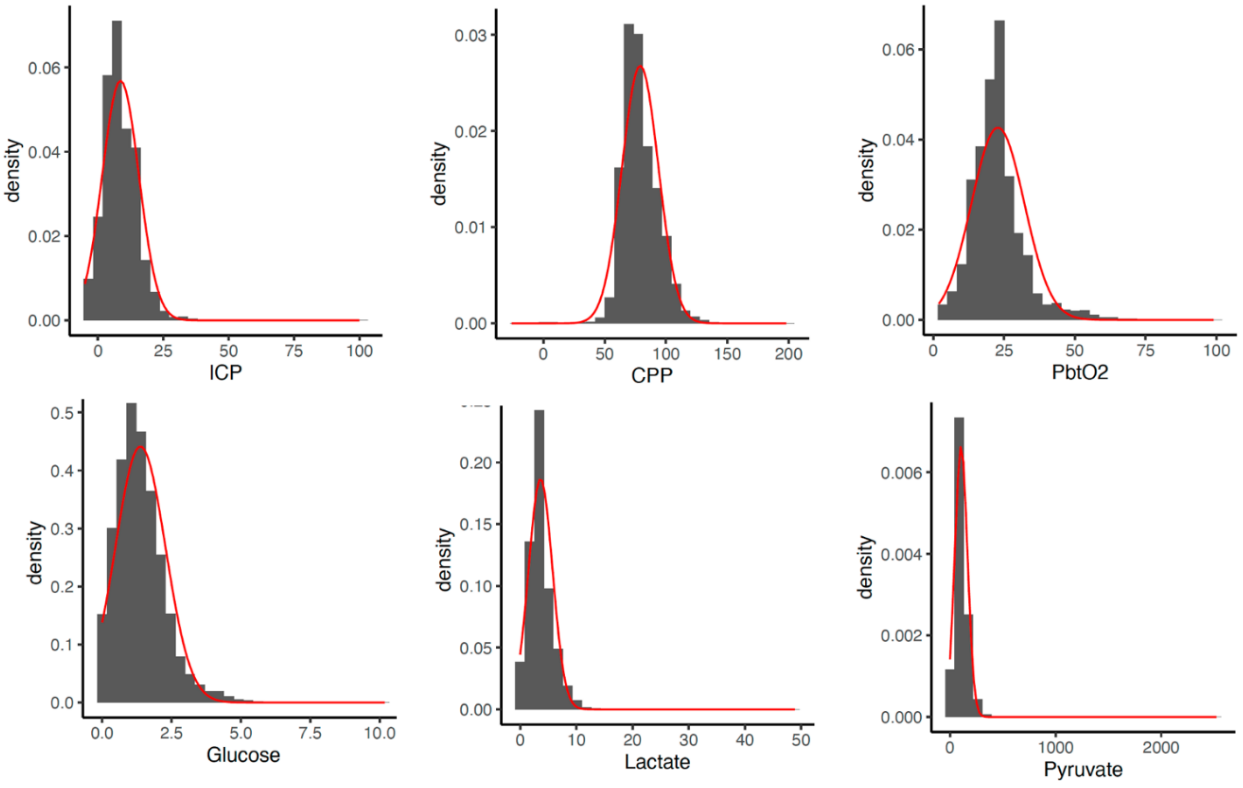

Figure legend: Histograms of the raw data distribution of each neuromonitoring parameter in the total patient population. Abbreviations: CPP = Cerebral Perfusion Pressure; ICP = Intracranial Pressure; $PbtO_2$ = Brain Tissue Oxygen Tension.

SUPPLEMENTARY FIGURE S2: CORRELATIONS BETWEEN NEUROMONITORING PARAMETERS

Patients with SAH (n=52):

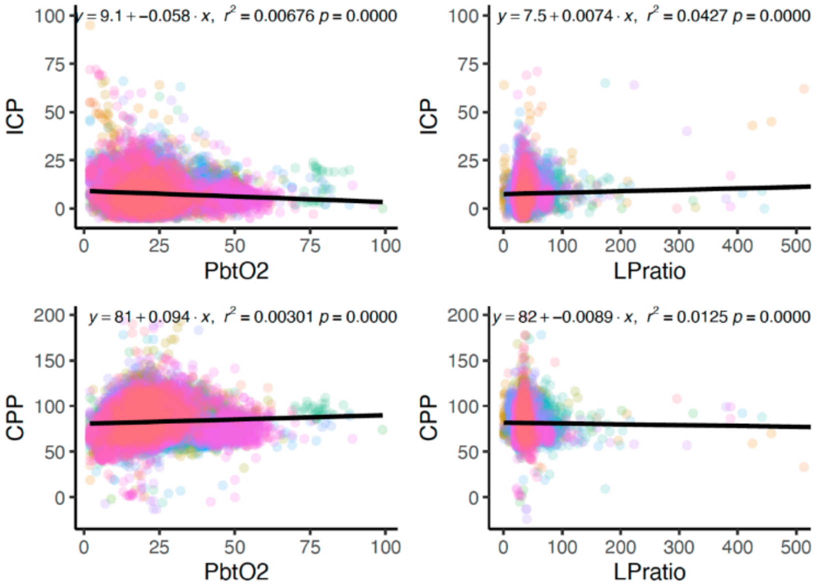

Patients with TBI (n=42):

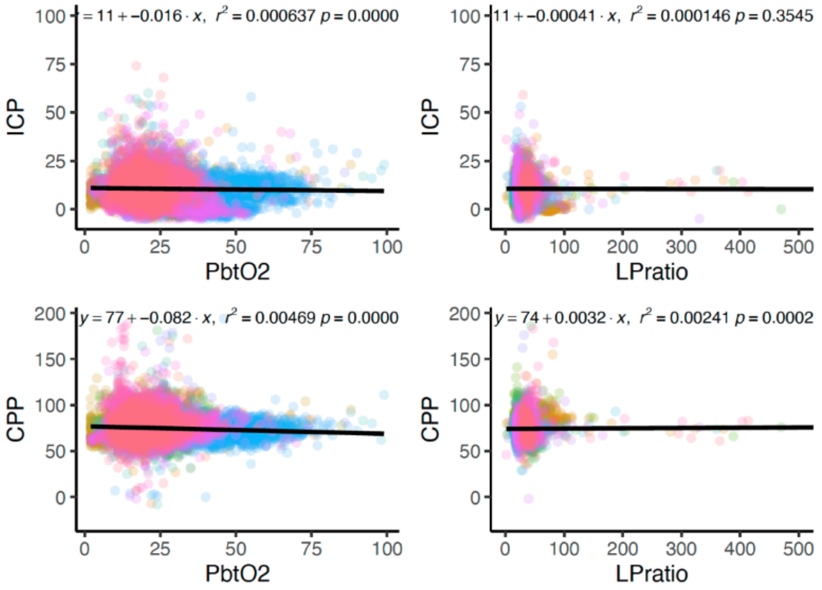

Figure legend: Linear regression lines and Pearsons correlation coefficients of the association between neuromonitoring parameters. Abbreviations: CPP = Cerebral Perfusion Pressure; ICP = Intracranial Pressure; LP-ratio = Lactate/Pyruvate Ratio; $PbtO_2$ = Brain Tissue Oxygen Tension; SAH = Subarachnoid Haemorrhage; TBI = Traumatic Brain Injury.

SUPPLEMENTARY FIGURE S3:

SUMMARY OF MAIN RESULTS

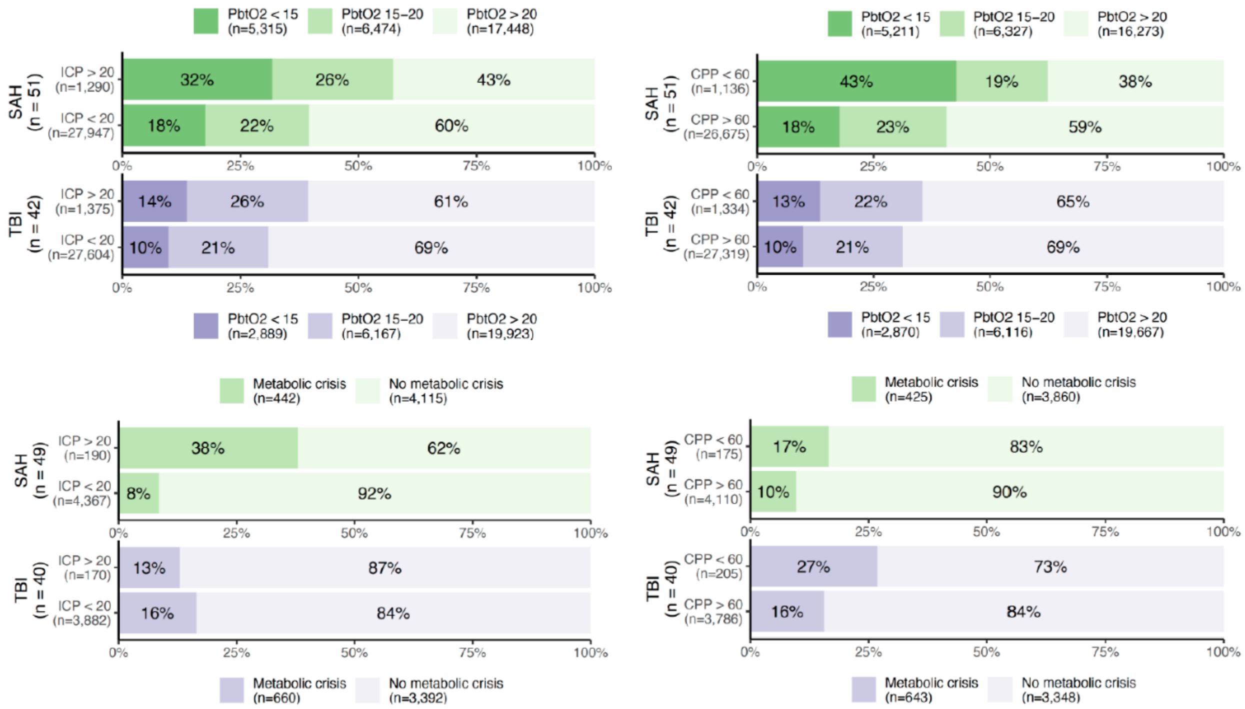

Figure legend: Relationships between ICP, CPP, $PbtO_2$ and brain metabolism. Figures are stratified by diagnosis (SAH or TBI), and the numbers and percentages displayed are "events" (i.e., concurrent measurements of the two neuromonitoring parameters). Elevated ICP was defined as >20 mmHg, and an abnormal CPP as <60 mmHg. Brain hypoxia was subdivided into mild ($PbtO_2$ 15-20 mmHg) and severe ($PbtO_2$ <15 mmHg). Metabolic crisis was defined as a lactate/pyruvate ratio >40 with a glucose level <0.2 mmol/L in cerebral microdialysate. **Abbreviations:** CPP = Cerebral Perfusion Pressure; ICP = Intracranial Pressure; $PbtO_2$ = Brain Tissue Oxygen Tension; SAH = Subarachnoid Haemorrhage; TBI = Traumatic Brain Injury.
